# Hyperosmolar Hyperglycemic State-Induced Hyperviscosity as a Catalyst for Multiple Embolic Strokes

**DOI:** 10.7759/cureus.63331

**Published:** 2024-06-27

**Authors:** Lorena Escaño, Prarthana Desai

**Affiliations:** 1 Internal Medicine, Danbury Hospital, Danbury, USA

**Keywords:** embolic stroke, diabetes mellitus, hyperglycemic emergency, prothrombotic state, hyperosmolar hyperglycaemic state

## Abstract

Hyperosmolar hyperglycemic state (HHS) is the most serious emergency in patients with uncontrolled diabetes mellitus. It has been associated with a prothrombotic state that increases the risk for ischemia in affected patients. Despite the literature on the risk of ischemic stroke in patients with chronic hyperglycemia being vast, there is not enough documentation on the risk of developing a stroke during a hyperglycemic crisis. We present a rare case of an 86-year-old male who was admitted with HHS whose hospital course was further complicated by multiple embolic strokes.

Prompt recognition of cerebral infarction when it intertwines with HHS remains a challenging task. This case emphasizes the value of clinical vigilance in patients with this hyperglycemic crisis. Further research is needed to better understand what this prothrombotic state truly entails in these patients.

## Introduction

Hyperosmolar hyperglycemic state (HHS) is one of the metabolic emergencies that can arise in decompensated diabetes mellitus. Despite well-developed diagnostic criteria and treatment protocols, it remains an important cause of mortality which approaches 20% in affected patients. Its danger lies in the fact that it typically impacts elderly patients with multiple comorbidities. In addition to this, it has a gradual onset, and the severe dehydration and increased plasma osmolality seen in these patients can ultimately lead to coma and death [1].

Multiple precipitating factors have been described for HHS, inadequate glycemic control and infection being the most common. However, some of the complications that can arise due to this metabolic emergency are less documented in the literature. In particular, the hypercoagulable state seen in affected patients can result in vessel thrombosis with subsequent myocardial infarction, cerebrovascular accident, pulmonary embolism or limb ischemia [2-5].

## Case presentation

We present an 86-year-old male brought to the emergency department after being found lying on his bathroom floor, profoundly lethargic and confused. Past medical history was remarkable for hypertension on amlodipine, heart failure with preserved ejection fraction on spironolactone and furosemide, and hyperlipidemia on atorvastatin. There was no known history of diabetes. 

The family at bedside reported the patient having a four-day history of decreased appetite with poor oral intake, generalized weakness, and increased urinary frequency. Symptoms continued to progress and the patient became lethargic and confused. EMS was called when the patient was found on his bathroom floor. 

Upon arrival to the emergency department, the patient was restless, confused, afebrile, blood pressure 137/65 mmHg, heart rate of 108 b/m, respiratory rate 24 r/m, saturating well on room air. Physical examination was only remarkable for mild epigastric tenderness. No focal neurological deficit was appreciated. 

Pertinent laboratory values on admission are presented in Table 1. Urinalysis showed 2+ glucose and trace ketones. 

**Table 1:** Pertinent laboratory results INR: international normalised ratio, PT: prothrombin time, PTT: partial thromboplastin time

Laboratory test	Laboratory result	Reference value
Glucose	>1500 mg/dl	70 – 99 mg/dl
Serum osmolality	354 mOsm/kg	275 – 295 mOsm/kg
Bicarbonate	19 mmol/L	22 – 29 mmol/L
Anion gap	24 mmol/L	8 – 18 mmol/L
Venous pH	7.23	7.32 – 7.43
Beta hydroxybutyrate	3.9 mmol/L	0 – 0.4 mmol/L
Lactic acid	3.6 mmol/L	0.5 – 2.2 mmol/L
Lipase	1,825 U/L	13 – 60 U/L
Triglycerides	420 mg/dL	0 – 149 mg/dL
Corrected sodium	153 mmol/L	135 – 145 mmol/L
Potassium	4.5 mmol/L	3.5 – 5.3 mmol/L
Creatinine	2.10 mg/dL	0.67 – 1.23 mg/dL
Glomerular filtration rate	30 mL/min	>60 mL/min
White blood cells	19,100	3,500 – 10,000
Platelet count	334,000	150,000 – 400,000
Hemoglobin	13.9	13.5 – 17
Hematocrit	44.1%	38 – 50
Hemoglobin A1C	12.7%	<5.7%
INR	1.12	0.91 – 1.40
PT	14.4	12.2 – 14.5
PTT	32.9	25 – 35.8

**Figure 1:**
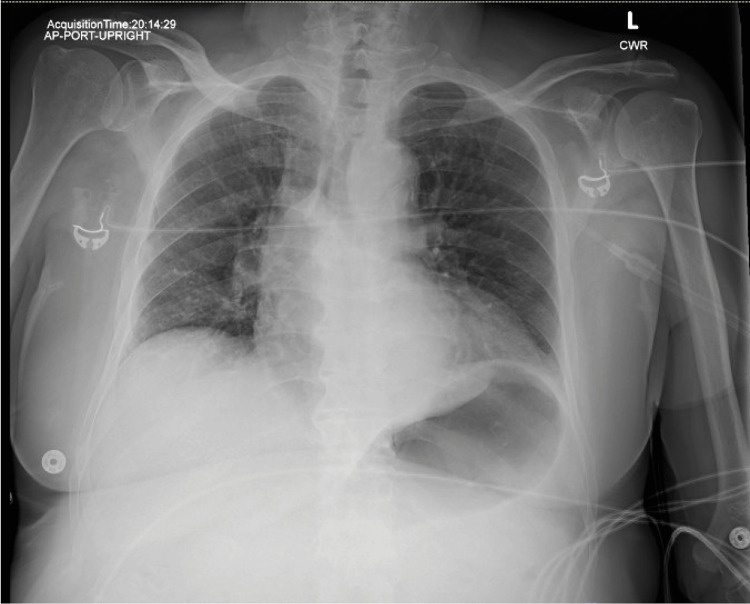
Anteroposterior chest x-ray on admission

**Figure 2:**
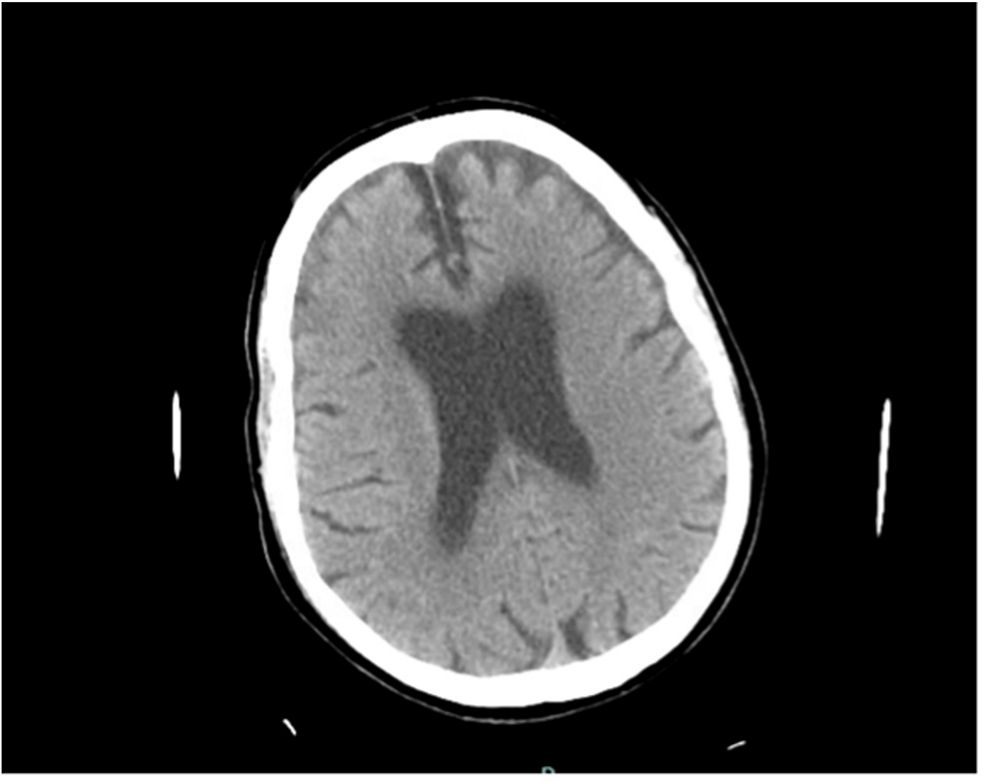
Computed tomography of the head on admission

**Figure 3:**
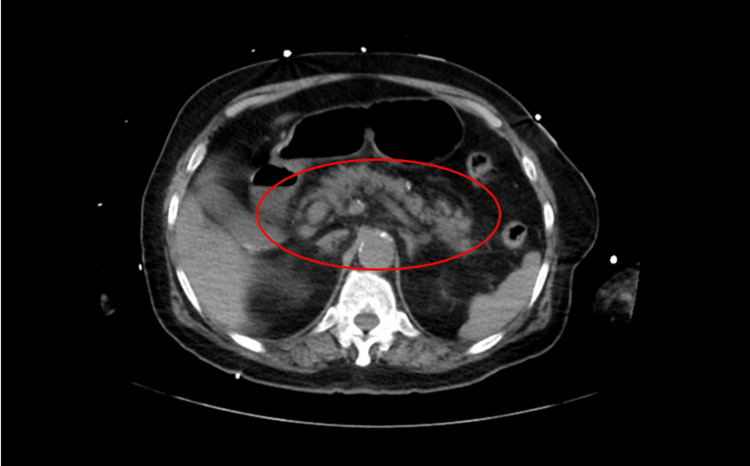
Computed tomography of the abdomen without contrast showing peripancreatic fat stranding on admission

Chest X-ray on admission showed no acute cardiopulmonary process (Figure 1). CT of the head was unremarkable for any acute intracranial abnormality (Figure 2). CT abdomen and pelvis showed peripancreatic fat stranding concerning for acute pancreatitis (Figure 3). Abdominal ultrasound was unremarkable for obstructive biliary disease. 

The patient was admitted with a working diagnosis of hyperosmolar hyperglycemic state with a component of diabetic ketoacidosis (DKA) and acute pancreatitis as the likely precipitant.

Given worsening mental status and tachypnea, the patient was intubated and sedated for airway protection. Initial management included IV fluids and insulin drip with subsequent improvement of glycemia. The rate of hyperglycemia correction is illustrated in Table 2. It was corrected at 40-80 mg/dl per hour as per protocol to avoid the development of cerebral edema. 

**Table 2:** Rate of correction of hyperglycemia in the first 24 hours

Glucose level on admission	>1500 mg/dl
Glucose level 4 hours post admission	1210 mg/dl
Glucose level 8 hours post admission	895 mg/dl
Glucose level 12 hours post admission	715 mg/dl
Glucose level 16 hours post admission	468 mg/dl
Glucose level 20 hours post admission	429 mg/dl
Glucose level 24 hours post admission	277 mg/dl

**Figure 4:**
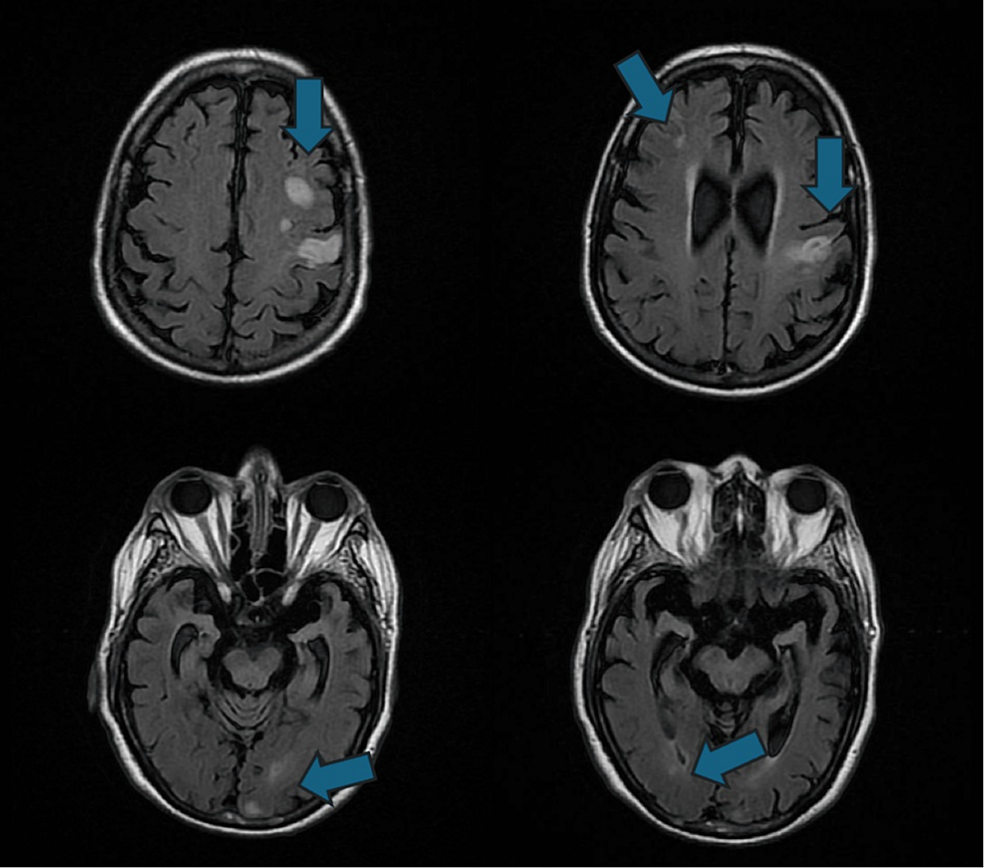
Magnetic resonance imaging of the brain. Axial T2 fluid attenuated inversion recovery (FLAIR) imaging

On day two of hospitalization, while weaning the patient off sedation, he was found to have new right-sided hemiparesis prompting further workup. MRI of the brain without contrast revealed multiple small acute infarcts within both frontal lobes, left frontoparietal region and occipital lobe affecting multiple vascular territories (Figure 4). Echocardiogram showed preserved ejection fraction without findings concerning for intracardiac thrombus. Carotid duplex was unremarkable. Cardiac monitoring was without evidence of atrial fibrillation. Neurology was consulted and the patient was started on Aspirin as per protocol. Home atorvastatin dose was increased. Prophylactic heparin which was initially held due to gastrointestinal bleed with dropping hemoglobin was also resumed. 

The patient’s poor mental status and neurological deficits persisted and started to improve very gradually despite correction of severe hyperglycemia. He was ultimately discharged to an acute rehabilitation facility to continue therapy for residual neurological deficits. The acute pancreatitis improved with conservative management without the need for further interventions. The patient was eventually transitioned to a subcutaneous insulin regimen with the plan to continue new-onset diabetes work-up as an outpatient. 

## Discussion

We have presented here a compelling case involving an 86-year-old male who was initially admitted with a hyperosmolar hyperglycemic state and a component of DKA, likely precipitated by acute pancreatitis, whose hospital course was further complicated by multiple embolic strokes.

Profound dehydration, significant hyperglycemia and hyperosmolarity with some degree of altered mentation, are all well-known features of HHS. However, less discussed in literature is that these metabolic derangements can lead to a prothrombotic state, further increasing the risk of ischemia in these patients. The severe hyperglycemia seen in HHS leads to significant osmotic diuresis which ends up in severe dehydration. This results in the concentration of blood constituents causing increased blood viscosity and stasis. Adding to the pathogenesis, the hyperglycemia and elevated osmolality precipitate endothelial cell dysfunction which promotes vasoconstriction. This cascade of events is further exacerbated by the release of coagulation factors and platelet hyperactivity, as well as stress-induced release of proinflammatory cytokines. Each one of these events results in a prothrombotic state that increases the risk for ischemia as seen in our patient [2].

The current guidelines for the management of HHS advise the use of prophylactic anticoagulation for the entire duration of the hospital stay, unless there are contraindications [4]. Our patient had a prolonged hospital stay that was complicated by an episode gastrointestinal bleed and downtrending hemoglobin, causing pharmacological prophylactic anticoagulation to be deferred. Once his hemoglobin stabilized and the benefits outweighed the risks, prophylactic anticoagulation was resumed. 

A few case reports have documented this prothrombotic state seen in HHS presenting as ischemic stroke, venous thrombosis, pulmonary embolism, myocardial infarction and even acute limb ischemia [2,4,5]. However, the occurrence of stroke as a complication of this hypercoagulable state is not that well detailed in the literature as HHS on its own can present as a stroke mimic with neurological deficits and even MRI abnormalities. Despite similar presentation, when HHS presents as a stroke mimic, complete resolution of symptoms is seen once severe hyperglycemia is corrected [3]. In view of this, when both HSS and ischemic stroke coexist, the diagnosis of the latter can be a clinical challenge and can be delayed. 

An intriguing aspect of this case was the development of new neurological deficits on day two of hospitalization and lack of resolution of symptoms once the severe hyperglycemia improved. This in addition to the MRI findings reinforce the diagnosis of hyperviscosity-induced multiple embolic strokes in the setting of hyperosmolar hyperglycemic state. 

## Conclusions

This case emphasizes the importance of recognizing this prothrombotic state with higher risk of ischemia as a potential complication of hyperosmolar hyperglycemic state. In addition to this, it highlights the clinical complexity of HHS and acute stroke when they coexist. This can lead to delayed diagnosis and management of a neurological disease that can lead to deleterious outcomes if left untreated, especially when time-sensitive interventions such as thrombolysis and thrombectomy are warranted.

Further research is needed to gain a deeper understanding of what this hypercoagulable state truly implies in these patients, including its characteristics and overall risk increment of ischemia.
